# Prosthesis for Unilateral Isolated Proximal Focal Femoral Deficiency: A Case Report and Review of the Literature

**DOI:** 10.7759/cureus.63210

**Published:** 2024-06-26

**Authors:** Kashif Akhtar Ahmed, Rajdeep Das, Kalyan Sarma, Bipul Kumar Das, Dibyajyoti Saikia

**Affiliations:** 1 Orthopaedics, All India Institute of Medical Sciences, Guwahati, Guwahati, IND; 2 Radiodiagnosis and Interventional Radiology, All India Institute of Medical Sciences, Guwahati, Guwahati, IND; 3 Paediatrics, All India Institute of Medical Sciences, Guwahati, Guwahati, IND; 4 Pharmacology, All India Institute of Medical Sciences, Guwahati, Guwahati, IND

**Keywords:** femur, congenital abnormality, extension prosthesis, prosthesis, proximal focal femoral deficiency

## Abstract

A 14-month-old female child was brought to us by her parents with the complaint of progressive shortening of her right lower limb since birth. Born to non-consanguineous parents from a poor socioeconomic background, her birth and antenatal history were uneventful. Physical examination revealed no facial dysmorphism; however, her right thigh was short and bulky, and there were restrictions in hip, knee, and ankle movements. The pediatric evaluation showed normal growth and development. X-rays confirmed proximal femoral focal deficiency (PFFD) of the right lower limb. After extensive parental counseling regarding the condition, potential interventions, and outcomes, the parents opted for prosthetic management due to concerns about surgical costs, risks, and cosmetic outcomes. A custom-made extension prosthesis was prepared for the limb and was fit. At a follow-up of one year, the child exhibited a normal gait pattern with stable hip, knee, and ankle motion, and the parents expressed satisfaction with the prosthetic management, preferring it over surgical intervention for the time being.

## Introduction

Proximal femoral focal deficiency (PFFD) is a very uncommon innate hypoplastic condition of the proximal portion of the thigh bone (femur), expressing as a shortened limb and bulky thigh. PFFD is sporadically inherited; nevertheless, a couple of familial cases exist in the literature [1]. The incidence of PFFD lies between 0.11 and 0.2 per million live births. Unilateral PFFD occurs most commonly in 85%-90% of patients [2]. In unilateral involvement, the right limb is more commonly involved [3].

The management of PFFD is not rigid or straightforward but is complex and multifactorial and regulated by several competing factors, including the extent of leg length discrepancy, the association of other anomalies, deficiency and/or deformity, musculature, the status of hip and knee joints, on-hand experience of the surgeon, accessibility or affordability of appropriate prostheses, and the expectations from the parents as well as the child, which are again ascertained by the sociocultural environment, economical and educational background, and the parent's capacity and/or tenacity to go through an expected complex treatment and long-drawn-out management [4,5]. In PFFD, the most obvious functional limitation is due to a shortened lower extremity, as mentioned previously; this provides scope for non-operative treatment of a child [6]. Here, we present a case of unilateral PFFD in a 14-month-old female child and describe her prosthetic management prior to any surgical procedure, with a short review of the relevant literature on prosthetic management of PFFD.

## Case presentation

**Figure 1:**
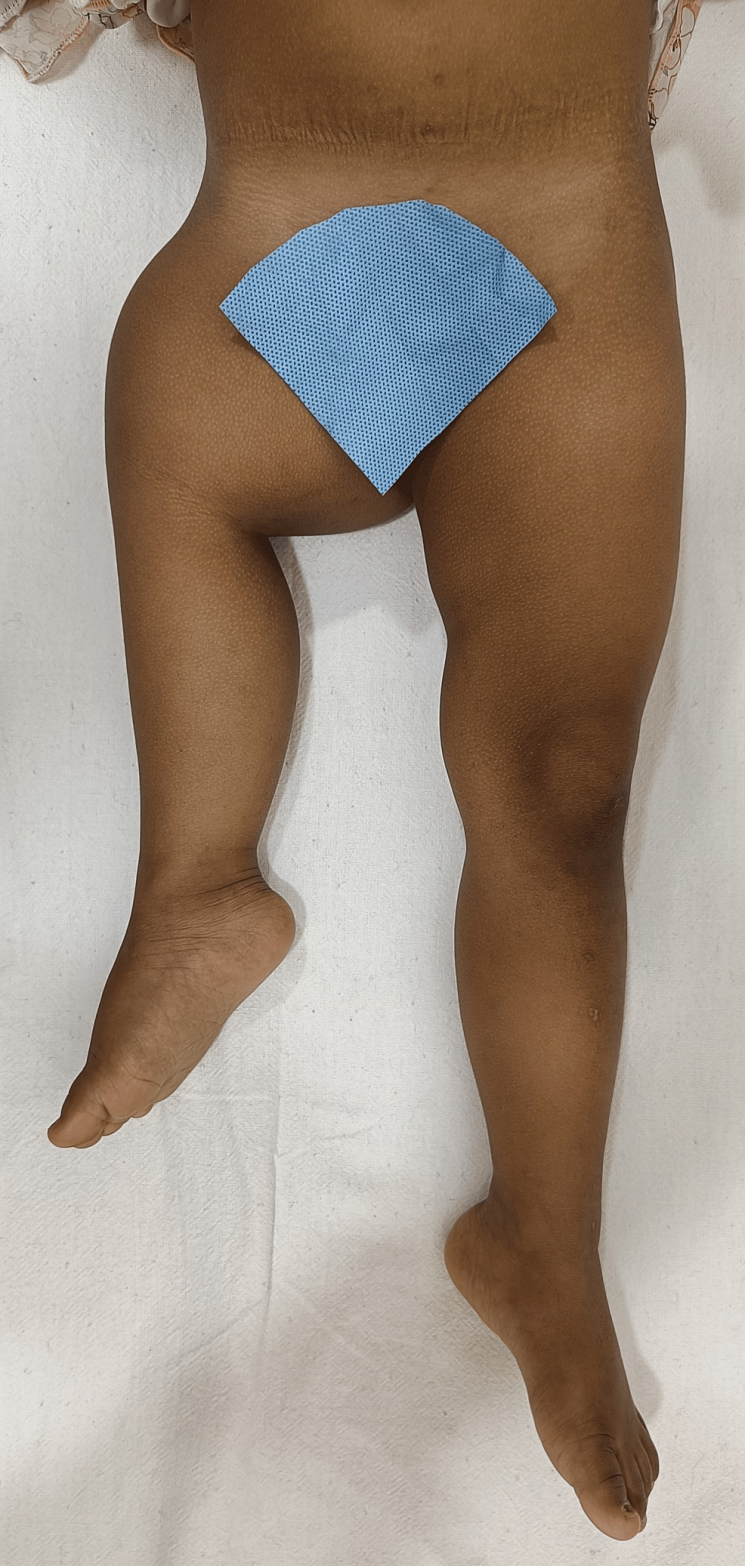
Clinical picture of the child showing the short right lower limb The right thigh is short and bulky with flexion, abduction, and external rotation of the right hip joint.

A 14-month-old female child was brought by her parents to our Orthopaedics Outpatient Department in March 2023. The parents presented that the child has a history of shortened right lower limb since birth with slowly progressive shortening with her growth after that (Figure 1).

She was the first child of her parents, consequent of a healthy non-consanguineous marriage. The father was 32 years old, and the mother was 28 years old at the child's birth. They belonged to poor socioeconomic status. The child's mother had a 39 weeks term spontaneous, uncomplicated normal vaginal delivery in vertex presentation of the baby. The mother's antenatal history was uneventful. However, an antenatal anomaly scan was not done for the mother. There was no history of maternal diabetes, gestational diabetes, intake of any drugs, injury, or trauma suffered by the mother during the pregnancy. The mother could not recollect any history of toxins exposure. There was no family history of similar occurrences among other family members and relatives.

**Figure 2:**
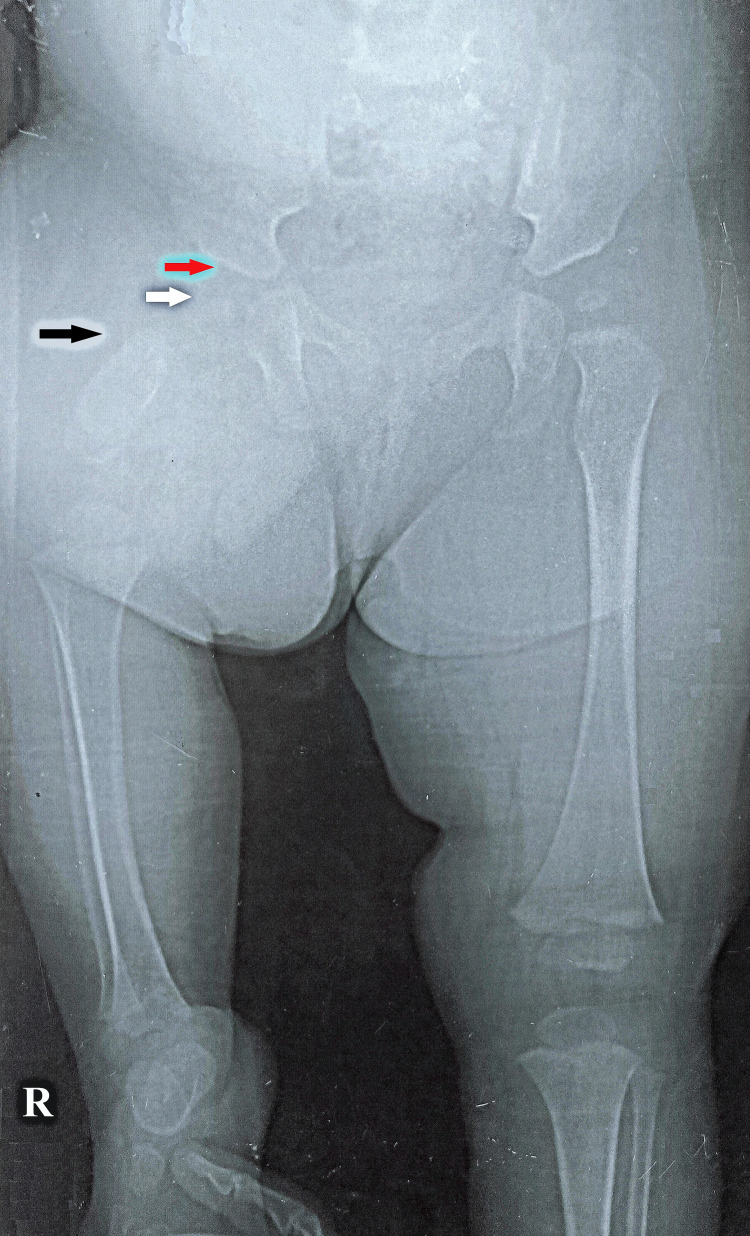
Anterior-posterior plain X-ray of the pelvis with the affected right leg and normal left thigh demonstrating a short right femur with deficiency of its proximal two-thirds (black arrow) The femoral head epiphysis (white arrow) as well as an adequate acetabulum (red arrow) is noted on the affected side, making it Aitken type A.

On physical examination, the child did not have any features of facial dysmorphism. The child's upper limbs and left lower limb were unremarkable. Her right thigh was bulky and short with external rotation, flexion, and abduction at the right hip. The right heel was 5 cm below the left knee joint line and 15 cm above the left heel. The movements at her right hip were slightly limited compared to her left hip, but there was a flexion deformity of 15° in right knee joint movements. Her right ankle joint had a 10° restriction of ankle dorsiflexion. A pediatric evaluation of the child was undertaken. The pediatric evaluation showed normal height, weight, and head circumference measurements. Neurological, cardiopulmonary, abdominal, and genital examinations were unremarkable. Her developmental milestones were normal for her age. The child could walk with the heel of the right limb and the knee of the left limb at presentation. A full-length lower extremity plain X-ray was done, which revealed Aitken type A (Figure 2) [7] and Gillespie and Torode [8] group 1 of PFFD of the right lower limb.

A detailed, meticulous discussion was done with the parents regarding the child's condition, the course of the deformity, possible interventions, and expected outcomes and limitations. Parental counseling sessions were organized with a multidisciplinary team composed of the orthopedic surgeon, pediatrician, physiotherapist, prosthetist, and orthotist. The parents were advised not to hurry about reaching a decision. They were informed about the life plan of the child with possible surgical interventions, the time duration of surgeries, estimated costs, possible risks, and expected final outcomes. It was empathetically emphasized that no significant distinction with respect to physical function and quality of life would be produced by prosthetics whether combined with or without surgical limb lengthening for their child.

**Figure 3:**
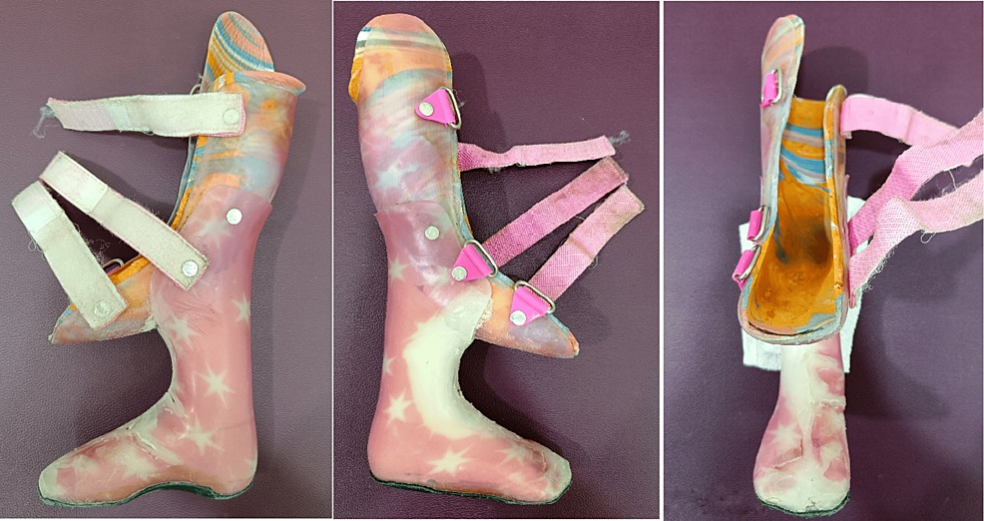
Extension prosthesis with artificial foot used for the child (side-to-side views and front view) The Velcro straps are used to fasten and keep the leg well-fitted in the prosthesis.

After two weeks, the parents reported back and opted for prosthetic management of the child for the present, as they continue to ponder about the possibility of surgeries in the future. The factors that were taken into consideration by the parents to opt against surgical correction at that point in time were the cost of surgical treatment, possible risks and complications, and the fact that full cosmetic correction may not be achieved. The child was fitted with a custom-made extension prosthesis with an artificial foot (Figure 3).

**Figure 4:**
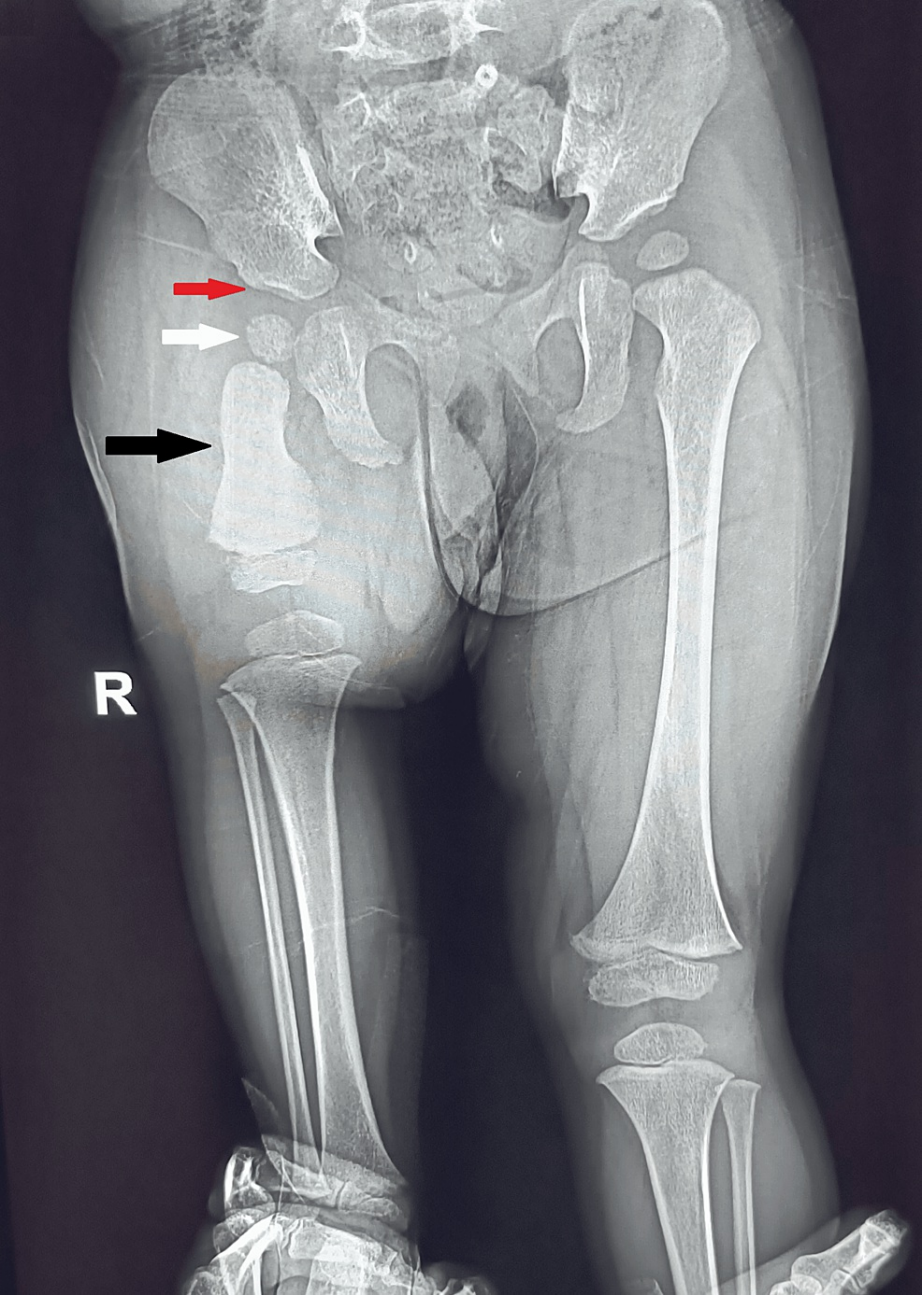
Anterior-posterior plain X-ray of the pelvis with the affected right leg and normal left thigh demonstrating a short right femur with deficiency of its proximal two-thirds (black arrow) The femoral head epiphysis (white arrow) within a well-formed acetabulum (red arrow) is noted on the affected side.

With a follow-up of one year, the child achieved a normal gait pattern, stable right hip joint, and normal right knee and right ankle motion as compared to the left side and no restriction of movements. The follow-up X-ray after one year of using the extension prosthesis with an artificial foot is shown in Figure 4. The parents are satisfied with the extension prosthesis and are presently not willing for surgical intervention.

## Discussion

Proximal focal femoral deficiency (PFFD) is an innate transverse deficiency condition in which the thigh bone (femur) is entirely shortened, associated with a typical hip deformity of the affected limb. It belongs to the spectrum of congenital femoral deficiency (CFD), which incorporates deficiency of the distal femur or hypoplastic or aplastic femur. The severity of affection caused by PFFD is wide, encompassing the degree of involvement of bone with surrounding soft tissues and neurovascular structures [1]. It frequently involves additive malformations of the distal part of the limb, giving rise to a variety of complex manifestations [9].

Various risk factors are documented in the literature for PFFD, such as ischemia, hypoxia, maternal diabetes mellitus, exposure to radiation, toxins, microbiological organisms, drugs such as thalidomide, and thermal or mechanical injuries, hormones, and injury to the fetus between the fourth to eighth weeks of gestation [10,11].

The Gillespie and Torode classification [8] is a simple classification for families and surgeons regarding the treatment of the affected limb. It rationalizes decision-making for surgical treatment. This classification correlates early clinical signs with long-term treatment plans [12]. The classification helps to clearly identify and differentiate patients who can benefit from limb-lengthening surgeries from patients who are better suited for non-surgical management with prosthetics [8]. The Gillespie and Torode classification is illustrated in Table 1.

**Table 1:** Features of the differentiation between Gillespie and Torode group 1 and group 2 patients as suggested by the Gillespie and Torode classification, elaborating the clinical and radiological differences between the two groups Reference: Gillespie R, Torode IP: Classification and management of congenital abnormalities of the femur. J Bone Joint Surg Br. 1983, 65:557-68. 10.1302/0301-620X.65B5.6643558 [8]

Features	Gillespie and Torode group 1 (congenital hypoplastic femur)	Gillespie and Torode group 2 (true proximal focal femoral deficiency)
Limb length discrepancy	The affected limb foot is around the middle of the normal opposite tibia	The affected limb foot is at or shorter than the level of the opposite knee
Proximal femur	There is no defect in the cartilage of the femoral head, neck, and greater trochanter, and the head and neck are in varus and retroversion, and coxa vara radiologically	True deficiency exists between the cartilaginous portion of the femoral head and the shaft of the femur, and the head and neck may be absent radiologically
Femur length	Short, 40%-60% of the opposite femur	Very short, 35%-50% of the opposite femur
Knee	Anteroposterior laxity, valgus, functional	Hypoplastic, marked anteroposterior laxity to congenital knee fusion, functionless
Flexion deformities of the hip and knee	Not severe, improves with age	Fixed contractures, do not improve with age
Goal of treatment	Limb length equalization may be possible, and surgery may be directed toward limb reconstructive lengthening	A prosthesis is a must, and surgeries should be directed to modifying the leg to achieve good fit prosthesis with satisfactory functional and cosmetic outcomes
Aitken type	Type A	Type B, C, and D

The Gillespie and Torode group 1 includes congenital short femur patients (Aitken type A), where limb length equalization should be the ultimate goal of treatment. The Gillespie and Torode group 2 includes the actual PFFD patients (Aitken type B, C, and D), who invariably require prosthetic management, and all surgeries must be planned to facilitate the fitting of prosthesis [8]. The likelihood of surgical reconstruction and limb lengthening is remote in group 2 patients.

The initial time for intervention in the management of a PFFD child concurs with the age of the child when the child has just started standing [6]. The management of PFFD requires integration between pediatric orthopedic surgeons, prosthetists, and physical therapists with the aim of equalizing the limb length with or without surgery, as early as the child starts walking (at 1-2 years) [13]. The fundamental principle is to initiate the therapy early to attain the capable length of the femur bone [14]. The advancing limb discrepancy frequently causes deterioration of gait, leading to subsequent consequences such as musculotendinous contractures and equinus deformity of the foot [9]. Hence, correct gait training with a prosthesis becomes imperative to establish hip and knee stability, equalize leg lengths, and avoid equinus deformity while standing and walking [6].

In a child with >50% shortening of limb or a predicted limb length discrepancy of >20 cm, treatment includes prosthesis use with or without surgical correction. Options for surgery include Syme amputation or modified Boyd amputation with or without knee arthrodesis, rotationplasty, and femoro-pelvic arthrodesis [15].

The treatment options for the use of prosthesis without surgical correction include Moseley patient-friendly functional device and hybrid prosthesis [16,17]. The advantages of Moseley device and unrotated ankle include (1) augmented stability of the knee, (2) significantly better gait consciousness and limb proprioception, (3) spontaneity of the use because the ankle joint is homologous to the opposite knee in function and posture with respect to gait cycle, and (4) physiological, mental, and social developmental rewards, regarding the sustenance of proper structural image of torso with lower extremity [16]. Hybrid prosthesis combines the technology of orthotics and prosthetics with their biomechanical principles, hence also known as "prosthosis." The patient can easily control the ground reaction forces that are more efficiently transferred to the limb. It increases stability, gait speed, and agility. A more normal gait pattern develops using the patient's muscles and enhanced proprioception. Moreover, length adjustments are also possible in hybrid prostheses [17]. The critically afflicted children tend to choose light foot-in-foot prosthesis (also known as extension prosthesis or extension prosthesis with an artificial foot) [9].

Prosthesis should be applied in all cases regardless of the ultimate treatment plan, whether surgical or non-surgical, and the first management must contemporize with normal development. It serves as a "stopgap" as we wait for the baby to attain the age for surgical interventions or amidst the interval of surgical reconstruction [18]. Moreover, for an appropriately selected patient, no significant distinction with respect to physical function and quality of life was appreciable between prosthetics and lengthening [19].

The flowchart of decisive factors for the treatment can be detailed to the child's parents by outlining timelines and clarifying expectations, thus creating a life plan [9]. We strongly believe that this holds true even in the setting of prosthetic management without surgical corrections. It is a delicate but baffling conversation to have with the parents and family of a child with PFFD, but the principles, expected goals, potential complications, and limitations of every treatment pathway should be elaborated, explained, and dissertated in an informed and balanced manner [4]. The conversation must be initiated early with vehemence of the point that a surgically lengthened limb need not always yield superior functional outcomes.

Prenatal diagnosis of PFFD is helpful to recognize the deformity early and provides parents and physicians with valuable information on management and future therapeutic planning [20]. Expertise and technical progressions in ultrasound imaging have resulted in prenatal diagnosis of congenital limb abnormalities and brought in the necessity for rational conversation at an unimaginably early phase of pregnancy [4]. The extent of shortening gets constant after the first trimester, which can allow a precise estimation of the amount of expected limb length discrepancy in adulthood. The treatment plan must be fostered and developed by a multidisciplinary team with the primary goal of achieving satisfactory functional outcomes [4]. Monsell et al. [4] suggested that the initial conversation must be focused on long-run quality of life and functional outcomes and never on surgical procedures.

## Conclusions

The management of PFFD in children requires a holistic approach encompassing psychological support, informed decision-making, timely interventions, and continuous monitoring. The decision must be individualized, considering the child's specific condition, growth patterns, and overall health. The use of prosthetics plays a crucial role in fulfilling the physical, mental, and social demands of children with PFFD. Prosthetics use also stimulates bone growth and adaptation, potentially reducing the severity of future complications. Technological advancements in prostheses are improving the quality of life constantly for children with PFFD. Innovations in materials, design, and functionality of prostheses allow for greater mobility, comfort, and adaptability. Ongoing research and development in this field promise even better outcomes in the future, offering children enhanced opportunities for active and fulfilling lives, underscoring the importance of a multifaceted treatment paradigm.
